# A Study of Certain Herbs Against Chlorpyrifos-induced Changes in Lipid and Protein Profile in Poultry

**DOI:** 10.4103/0971-6580.75854

**Published:** 2011

**Authors:** P. Bharathi, A. Gopala Reddy, A. Rajasekher Reddy, M. Alpharaj

**Affiliations:** Department of Pharmacology and Toxicology, College of Veterinary Science, Hyderabad - 30, India; 1 College of Veterinary Science, Hyderabad - 30, India; 2 Department of Pharmacology and Toxicology, College of Veterinary Science, Tirupati, Andhra Pradesh, India

**Keywords:** Broilers, chlorpyrifos, lipid profile, protein profile

## Abstract

A total of 225 male broiler chicks (*Cobb* strain) of day-old age were procured for the study. The chicks were randomly divided into 15 groups consisting of 15 chicks in each group. Group 1 was maintained as basal diet control and group 2 on chlorpyrifos (CPS) at 100 ppm in feed throughout 6 wk as iron toxic control without any treatment. Groups 3-15 were maintained on CPS at 100 ppm in feed for the 4 wk (28 days) of study and thereafter administered with different herbs and their combinations for remaining 2 wk. The blood samples were drawn from wing vein on 28^th^ day and 42^nd^ day from the birds in each group for the estimation of lipid and protein profiles. The birds were sacrificed at the end of 6^th^ week and liver tissues were collected for histological examination. The concentrations of total cholesterol, low density lipoprotein (LDL) cholesterol, triglycerides, total protein, albumin and globulins and the A/G ratio were increased significantly (*P*<;0.05) in toxic groups (2–15), while high density lipoprotein (HDL) cholesterol was significantly (*P*<;0.05) decreased at the end of 4^th^ week. However, following supplementation of herbs and herbal combinations, the values of lipid and protein profile in groups 3–15 revived toward normal at the end of 6^th^ week. Histopathology of liver in CPS toxic control (group 2) revealed areas of degeneration, while groups 3–15 that were treated with herbs and their combinations exhibited these changes in a milder form, indicating regenerative alterations. The study revealed that chorpyrifos-induced changes in lipid and protein profile were improved by supplementation of certain herbs.

## INTRODUCTION

Chlorpyrifos (CPS) is used in controlling a variety of insects, flees, termites, lice etc. It is used as an insecticide on grain, fruit nut and vegetable crops. Residual amounts of CPS are detected in water, soil, fabric and on surfaces for months to years. Goel *et al*.,[1] reported that CPS induces toxicity by generating free radicals and by altering the levels of enzymatic and non-enzymatic antioxidant defenses. Several herbs, spices and many Indian medicinal plants are considered to be potential sources of antioxidants.[2] Therefore, the present study was planned on broilers, using certain herbs and herbal combinations against CPS-induced toxicity with reference to lipid and protein profile.

## MATERIALS AND METHODS

A total of 225 sexed male broiler chicks (*Cobb* strain) of a day-old age were procured for the study. The chicks were randomly divided into 15 groups consisting of 15 chicks in each group. All the birds were provided with feed and water *ad libitum* throughout the experiment. All the groups were maintained as per the following treatment schedule for 6 wk:

Group 1: Basal diet control (1-42 days)
Group 2: Chlorpyrifos (100 ppm) toxic control (1–42 days)
Group 3: Chlorpyrifos (1–28 days) + *Withania somnifera* at 0.1% of feed (29–42 days)
Group 4: Chlorpyrifos (1–28 days) + *Ocimum sanctum* at 0.1% of feed (29–42 days)
Group 5: Chlorpyrifos (1–28 days) + *Asparagus racemosus* at 0.1% of feed (29–42 days)
Group 6: Chlorpyrifos (1–28 days) + *Andrographis paniculata* at 0.1% of feed (29–42 days)
Group 7: Chlorpyrifos (1–28 days) + *Murraya koenigii* at 0.1% of feed (29–42 days)
Group 8: Chlorpyrifos (1–28 days) + shilajit at 0.1% of feed (29–42 days)
Group 9: Chlorpyrifos (1–28 days) + *Gymnema sylvestre*at 0.1% of feed (29–42 days)
Group10: Chlorpyrifos (1–28 days) + *Allium sativum* at 0.1% of feed (29–42 days)
Group 11: Chlorpyrifos (1–28 days) + spirulina at 0.1% of feed (29–42 days)
Group 12: Chlorpyrifos (1–28 days) + ginseng at 0.1% of feed (29–42 days)
Group 13: Chlorpyrifos (1–28 days) + *Withania somnifera + Asparagus racemosus + Andrographis paniculata* at 0.05% of each in feed (29-42 days)
Group 14: Chlorpyrifos (1-28 days) + *Withania somnifera* + *Murraya koenigii* + *Allium sativum* at 0.05% of each in feed (29+42 days)
Group 15: Chlorpyrifos (1-28 days) + spirulina + shilajit + *Gymnema sylvestre* at 0.05% of each in feed (29-42 days)

Group 1 was negative control and group 2 was toxic control throughout the study. Groups 3–15 were toxic controls up to the end of 4^th^ week (1–28 days), and subsequently, these were kept on treatment with herbs and herbal combinations for the next two weeks (29–42 days). The blood samples were drawn from wing vein at the end of 4^th^ and 6^th^ weeks for the estimation of lipid and protein profile, by using commercially available diagnostic kits (Qualigens Pvt. Ltd., Mumbai, India). The birds were sacrificed at the end of 6^th^ week and liver tissues were collected for histological examination. The data were subjected to statistical analysis by applying one-way analysis of variance (ANOVA) using statistical package for social sciences (SPSS) 10^th^ version. Differences between means were tested using Duncan’s multiple comparison test and significance was set at *P*<;0.05.

## RESULTS AND DISCUSSION

The concentrations of total cholesterol, low density lipoprotein (LDL) cholesterol and triglycerides (mg/dl) recorded at the end of 4^th^ week in the basal diet control (group 1) were 128.78±0.67, 70.11±1.11 and 31.83±1.29, respectively, which were significantly (*P*<;0.05) increased in the CPS toxic controls (groups 2–15) with the values ranging from 143.77±0.49 to 147.81±1.21, from 94.73±0.60 to 98.56±1.13 and from 41.01±0.99 to 42.50±0.64, respectively. Following supplementation with herbs in groups 3–15, there was a significant (*P*<;0.05) decrease in the cholesterol levels at the end of 6^th^ week with the values ranging from 170.25±0.53 to 181.00±1.99, from 102.59±1.30 to 114.06±3.24 and from 47.91±0.76 to 55.75±3.52, respectively, as compared to the CPS toxic control group 2 (195.51±1.48, 133.08±1.31 and 67.52±1.60, respectively). However, these values were significantly (*P*<;0.05) higher when compared to the basal diet control (162.90±3.44, 92.02±3.23 and 31.83±1.29, respectively). The high density lipoprotein (HDL) cholesterol concentration (mg/dl) recorded at the end of 4^th^ week in the basal diet control (group 1) was 52.31±0.71, which was significantly (*P*<;0.05) decreased in CPS toxic controls (groups 2–15) with the values ranging from 40.03±0.38 to 40.98±0.13. Following supplementation with herbs in groups 3–15, there was significant (*P*<;0.05) increase in the HDL cholesterol concentration at the end of 6^th^ week (values ranged from 55.82±2.28 to 60.42±0.83) as compared to their corresponding 4^th^ week values and the CPS control group 2 (31.83±1.29). However, the groups 5–8 and 10–15 showed no significant difference as compared to the basal diet control (62.15±0.86), but the remaining groups showed significantly (*P*<;0.05) lower levels of HDL cholesterol.

An increase in the total cholesterol, triglycerides and LDL with a decrease in HDL in serum serves as an index for hepatopathy, cardiac damage as well as renal failure.[3] In the present study, concentrations of the total cholesterol, LDL and triglycerides were significantly increased in toxic control with a significant decrease in HDL concentration as compared to the basal diet control group. All these findings can be further substantiated from the histopathology and biomarker studies of liver and kidney. Treated groups showed significant decrease in total cholesterol, LDL and triglycerides, with a significant increase in the HDL concentration. The beneficial effects of herbs in test could be attributed to their lipid lowering property. This effect of ginseng may be attributed to the ginsenosides and other active principles. *A. sativum* decreases hepatic 3-methylglutaryl-COA reductase, cholesterol-7-hydroxylase and fatty acid synthetase, thus lowering the serum lipid profile.[4] Spirulina significantly increases lipoprotein lipase activity and thus reduces the serum cholesterol.[5] *M. koenigii* has tertiary and quaternary alkaloids, flavonoids and glycoside components that reduce lipid levels in animals.[6] *G. sylvestre* increases lecithin-cholesterol acyl transferase activity and thus decreases serum cholesterol.[7]

The concentrations of total protein, albumin and globulins (g/dl) recorded at the end of 4^th^ week in the basal diet control (group 1) were 3.50±0.140, 1.92±0.041 and 1.58±0.168, respectively, which were significantly (*P*<;0.05) decreased in the CPS toxic controls (groups 2-15) with the values ranging from 1.19±0.084 to 1.28±0.045, from 0.49±0.007 to 0.51±0.008 and from 0.67±0.077 to 0.78±0.051, respectively. However, following supplementation with herbs and herbal combinations in test, there was a significant (*P*<;0.05) increase in the total protein concentration in groups 3-15 at the end of 6^th^ week with the values ranging from 4.16±0.011 to 4.70±0.026, from 2.28±0.022 to 2.79±0.014 and from 1.83±0.048 to 1.96±0.043, respectively, as compared to their corresponding 4^th^ week values and toxic control group 2 (1.94±0.015, 0.96±0.019 and 0.98±0.024, respectively). Amongst all the groups, the protein profile remained significantly (*P*<;0.05) higher in the basal diet control (4.95±0.030, 3.00±0.018 and 1.94±0.046, respectively). The A/G ratio recorded at the end of the 4^th^ week in the basal diet control (group1) was 1.28±0.145, which was significantly (*P*<;0.05) decreased in CPS toxic controls (groups 2–15) with the values ranging from 0.64±0.041 to 0.79±0.065. Following supplementation with herbs in groups 3–15, there was a significant (*P*<;0.05) increase at the end of 6^th^ week with the values ranging from 1.20±0.026 to 1.52±0.055 as compared to their corresponding 4^th^ week values and CPS toxic control group 2 (0.98±0.042).

The protein profile was studied by assessing total protein, albumin, globulin and A/G ratio in serum of birds. They serve as indicators for hepatic damage. The study revealed a significant decrease in the concentrations of total protein, albumin and globulin and the A/G ratio in CPS toxic control group, which could be attributed to the reduced capacity of the liver to synthesize them. This could be due to the peroxidative damage of liver, which is the exclusive site of protein synthesis.[3] The altered protein profile may be due to the hepatic damage caused by oxidative stress and this is substantiated from the histological sections of liver in CPS toxic control group, which showed marked central vein congestion, hydropic degeneration, mild bile duct hyperplasia and dilated sinusoidal spaces with congestion in the sinusoidal spaces, whereas the groups supplemented with herbs showed regenerative changes in liver and improvement in protein profile, suggesting the therapeutic potential of the herbs in test.

In conclusion, the results of the present investigation show that CPS exerted alterations in lipid and protein profile along with histological alterations in liver. Use of herbs and herbal combinations in test could counter the adverse effects of CPS pre-induced toxicity to a major extent, suggesting their beneficial effects.

## References

[1] Goel A, Dani V, Dhawan DK (2005). Protective effects of zinc on lipid peroxidation, antioxidant enzymes and hepatic histoarchitecture in chlorpyrifos induced toxicity. Chem Biol Interact.

[2] Farrukh A, Iqbal A, Zafar M (2006). Antioxidant and free radical scavenging properties of twelve traditionally used Indian medicinal plants Turkish J Biol.

[3] Kaneko JJ, Harvey JW, Michael LB (1997). Clinical Biochemistry of Domestic Animals.

[4] Qureshi AA, Din ZZ, Abuirmeileh N, Burger WC, Ahmad Y, Elson CE (1983). Suppression of avian hepatic lipid metabolism by solvent. extracts of garlic: Impact on serum lipids. J Nutr.

[5] Iwata I (1990). Effects of spirulina on plasma lipoprotein lipase activity in rats. J Nutr Sci Vitaminol.

[6] Vinuthan MK, Girish Kumar V, Narayanaswamy M, Veena T (2007). Lipid lowering effect of aqueous leaves extract of *Murraya koenigii* (Curry leaf) on alloxan induced male diabetic rats. Pharmacog Mag.

[7] Shigemastsu N, Asano R, Shimosaka M, Okazaki M (2001). Administration with the extract of *Gymnema sylvestre* R. Br leaves on lipid metabolism in rats. Biol Pharmaceutical Bull.

